# Evaluation of the cardiac effects of norepinephrine and dobutamine in rats with septic shock

**DOI:** 10.1186/cc12979

**Published:** 2013-11-05

**Authors:** Ronald PM Goncalves, Jamil Assreuy, Jose E da Silva-Santos

**Affiliations:** 1 Department of Pharmacology, Universidade Federal de Santa Catarina, Florianópolis, Brazil

## Background

Hypotension and cardiac dysfunction are frequently found in severe sepsis and septic shock. Vasoactive and inotropic drugs are largely used to reverse hypotension, but its effects on heart function have been scarcely investigated [1]. We thus evaluated the influence of both norepinephrine and dobutamine on the cardiovascular function of rats subjected to cecal ligation and puncture (CLP).

## Materials and methods

The measurement of the cardiac function was performed in male Wistar rats (3 to 4 months old), kept under isoflurane-induced anesthesia (1 to 3%), using a pressure-volume catheter, which was inserted into the left ventricle through the carotid artery. Blood samples were collected from all animals for hematological analyses. The experiments were conducted at 24 and 48 hours after CLP. For this, the cecum was ligated with a ratio of 50% and perforated with a needle (18 G, four holes; mortality rate ~50% after 48 hours), followed by four subcutaneous injections (12/12 hours) of sterile saline (30 ml/kg) and tramadol (5 mg/kg), for fluid resuscitation and analgesia, respectively. Data were recorded at baseline and after single bolus administration of norepinephrine (1, 3 and 10 nmol/kg, i.v.) or dobutamine (3, 10 and 30 nmol/kg, i.v.). The results obtained in CLP groups were compared with control (CT) animals, which did not undergo any manipulation.

## Results

Both CLP 24 and 48 hour groups presented thrombocytopenia (~40% reduction), lymphopenia, hypoglycemia and leukopenia (*P *< 0.05), a clear indication of severe sepsis. However, only CLP 48 hour animals displayed refractory hypotension (MAP = 59 mmHg, vs. 78 mmHg in CT; *P *< 0.05) in spite of volume resuscitation. The highest doses of norepinephrine and dobutamine increased the MAP to 133.8 ± 8.1 and 97.8 ± 3.1 mmHg in CT, and to 120.6 ± 6.7 and 77.3 ± 4.4 mmHg in CLP 48 hour animals, respectively. The heart rate was significantly increased by norepinephrine and dobutamine in control, but not in CLP 48 hour animals. In addition, the basal values of both dP/dt_max _and dP/dt_min_, as well as after 1 nmol/kg dobutamine, were reduced in CLP 48 hour animals.

## Conclusions

Using a pressure-volume catheter in a closed-chest approach we demonstrated that, in spite of the ability to increase blood pressure, the chronotropic effects of norepinephrine and dobutamine are reduced at 48 hours after CLP in rats subjected to CLP. In addition, all doses of norepinephrine, but only by the highest doses of dobutamine, improved systolic and diastolic function in these animals.
